# Potential Phototoxicity of Indocyanine Green in Retinal Pigment Epithelial Cells after Angiography under Ambient Illumination

**DOI:** 10.1155/2018/6065285

**Published:** 2018-06-27

**Authors:** Tomohito Sato, Yoko Karasawa, Sho Ishikawa, Manzo Taguchi, Tadashi Muraoka, Masataka Ito, Masaru Takeuchi

**Affiliations:** ^1^Department of Ophthalmology, National Defense Medical College, Tokorozawa, Saitama, Japan; ^2^Department of Developmental Anatomy and Regenerative Biology, National Defense Medical College, Tokorozawa, Saitama, Japan

## Abstract

Indocyanine green (ICG) angiography is an indispensable inspection to diagnose and treat for chorioretinal diseases. In this study, we investigated the phototoxicity of ICG on RPE cells at the levels of residual ICG after angiography under ambient light. After incubation of ARPE-19 cells in a colorless medium containing 0 to 10 *μ*g/mL ICG for 24 hours in the dark or under 2000 lx illumination from a fluorescent lamp, cell viability decreased and cell death rate increased in cultures with more than 5.0 *μ*g/mL ICG under illumination. In culture with 10 *μ*g/mL ICG under illumination, morphology of cells changed to be oval and TUNEL- and malondialdehyde-positive cells increased compared to other cultures with ICG in the dark or without ICG under illumination. Furthermore, the level of intracellular reactive oxygen species was also elevated. On the other hand, toxicity of ICG denatured by illumination was not observed. Blocking green to red light overlapping wavelengths of ICG absorbance exhibited decreased cell death rate. The present study indicated that ICG at the estimated intravenous concentrations after ICG angiography induces potential phototoxicity on human RPE cells via oxidative damage under continuous ambient illumination and that the cytotoxicity is reduced by blocking green to red light wavelengths.

## 1. Introduction

Indocyanine green (ICG) is a photosensitive tricarbocyanine dye with peak spectral absorbance at approximately 780 nm [1, 2]. In clinical settings, ICG is used as an intravenous contrast medium to examine plasma volume, cardiac output, and hepatic functions as well as choroidal circulation in the eyes [3–5]. When injected intravenously, ICG binds to serum proteins forming ICG-protein complexes which are promptly excreted from systemic circulation into bile via hepatocytes [3, 6, 7]. ICG is generally acknowledged as a dye that has a low incidence of side effects [8]. On the other hand, ICG extravagates into the choroidal stroma and accumulates within the retinal pigment epithelium- (RPE-) Bruch's membrane complex and remains there for at least 24 hours after ICG angiography [9–12].

ICG angiography has been an indispensable inspection to diagnose and treat for chorioretinal diseases such as age-related macular degeneration (AMD) and uveitis in which fluorescein angiography is unable to define pathological lesions of choroidal vasculatures, accurately [13–15]. On the other hand, ICG toxicity on RPE which constitutes the outer blood-retinal barrier has been extensively examined *in vitro*, *in vivo*, and ex vivo, and several mechanisms of the toxicity were reported [16–24]. Liu et al. showed that 0.05 mg/mL ICG exposure for 3 hours induced a transient decrease in the tightness of tight junctions in cultured human foetal RPE cells [20]. In addition, ICG was uptaken by RPE cells *in vitro* and had toxic effects on RPE cells in dose- and exposure time-dependent manners [23–25].

ICG absorbs light energy, and the absorbed energy is converted to heat (photothermal effect) or transferred to molecular oxygen (type II photooxidation) generating singlet oxygen as a kind of reactive oxygen species (ROS) [1, 2, 26]. Kernt et al. reported that 0.05% exposure of ICG for 10 minutes decreased cell viability and slightly elevated the intracellular ROS level in primary human RPE cells, and irradiation by light pipe enhanced these effects in the cells with pre-ICG exposure [27]. Engel et al. showed that light-induced decomposition products of ICG had cytotoxic effects in porcine RPE cells [28].

Molecules of ICG in solution form monomer and dimer/oligomer dependently on its concentration and solvent medium [2]. The peak absorbance wavelengths of monomer are between 780 and 810 nm, and those of dimer and/or oligomer are between 680 and 730 nm [24]. Gandorfer et al. reported that the irradiation of light pipe at wavelengths from 620 to 760 nm, overlapping the wavelengths of ICG absorbance, caused severe inner retinal damage in ICG-exposed cadaveric eyes, whereas the damage was mild at wavelengths from 380 to 620 nm [22]. We previously reported that continuous illumination from a fluorescent lamp imitating ambient light caused phototoxicity after brief ICG exposure at high concentrations in cultured quail Müller cells and cultured human RPE cells, and the phototoxicity could be prevented by blocking the wavelengths of red light [19, 29]. On the other hand, ICG phototoxicity was primarily examined under particular conditions such as brief ICG exposure at high concentrations under intense irradiation by light pipe in these reports [22, 24, 28]. It has not been investigated for phototoxicity of ICG in RPE cells under ambient light at the levels of residual ICG concentrations after ICG angiography.

In the present study, we used a cell culture system equipped with a fluorescent lamp mimicking ambient light and found that 10 *μ*g/mL ICG regarded as almost the same as intravenous ICG concentration just after ICG angiography could induce cytotoxicity via oxidative damage under continuous irradiation of ambient light in human RPE cells. Furthermore, blocking green to red light could clearly prevent the ICG phototoxicity in the *vitro* model.

## 2. Results

### 2.1. Changes of Cell Death Rate in Culture with ICG under Illumination

**Figure 1:**
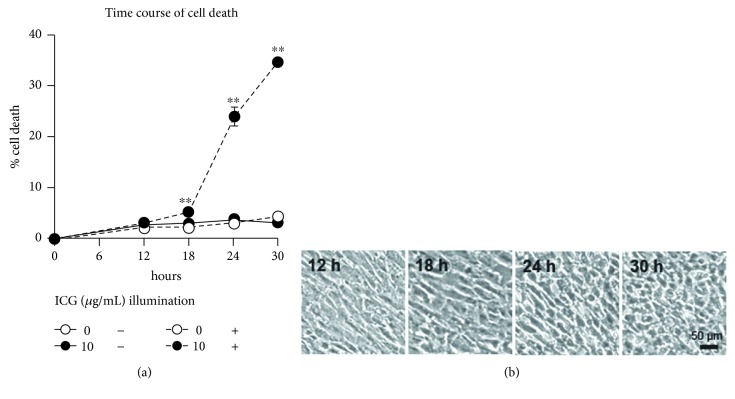
Time course of cell death rate and morphological changes cultured in the dark or under illumination. (a) Percentages of dead cells were measured by LDH activities in culture supernatants and cell lysates at indicated periods. The values of cell death in experimental cultures were compared with those in culture with 0 *μ*g/mL ICG in the dark. (b) Phase-contrast micrographs of cultures with 10 *μ*g/mL ICG under illumination at indicated periods. Dunnett's test: ^∗∗^*P* < 0.01.

To determine the most appropriate time for evaluating the effects of ICG and/or the illumination, we examined the time course of cell death rate in the cells cultured in the PBS-based medium with or without 10 *μ*g/mL ICG in the dark or under illumination (Figure 1(a)). In the culture without ICG in the dark or under illumination as well as the culture without ICG under illumination, the cell death rate increased slightly up to 30 hours. In the culture with ICG under illumination, the cell death rate increased gently until 18 hours, followed by a rapid increase up to 30 hours. When cell morphology was observed chronologically in the culture with ICG under illumination (Figure 1(b)), the cells remained flattened at 12 hours and 18 hours, changed to be round-shaped at 24 hours, and exhibited oval or shrunken appearance at 30 hours. Based on these results, we selected 24 hours as the most appropriate time for evaluating ICG phototoxicity.

### 2.2. Cellular Damages in Cultures with ICG under Illumination

**Figure 2:**
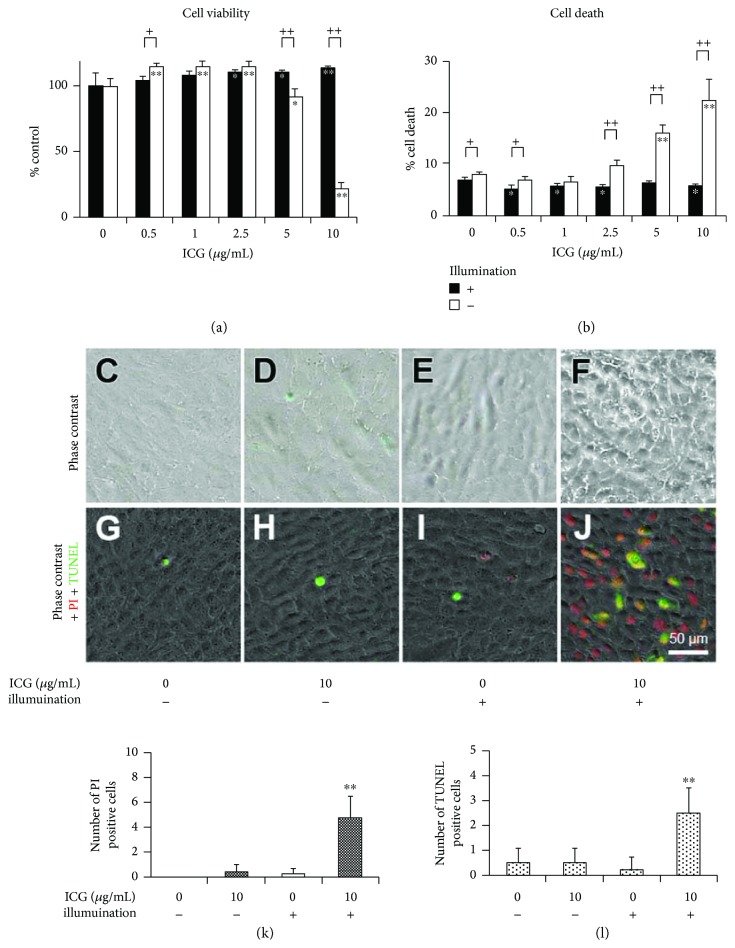
Phototoxicity of ICG exposed to RPE cells under illumination. Cells were cultured with 0 to 10 *μ*g/mL ICG in the dark or under illumination for 24 hours. (a) Cell viability and (b) cell death rate are shown. Cell viability of each culture measured by MTS assay was normalized to that of 0 *μ*g/mL ICG. (c–f) Phase-contrast micrographs. (g, h) Fluorescence micrographs of PI staining and TUNEL. Quantitation of PI-positive cells (k) and TUNEL-positive cells (l) in 10,000 *μ*m^2^. At least four squares of each experiment were analyzed. Dunnett's test: ^∗^*P* < 0.05 and ^∗∗^*P* < 0.01, unpaired Student's *t*-test: ^+^*P* < 0.05 and ^++^*P* < 0.01.

To examine influences of continuous ICG exposure, cell viability and cell death rate were measured in cultures containing 0 to 10 *μ*g/mL ICG after a 24-hour incubation in the dark or under illumination (Figures 2(a) and 2(b)). At 0 *μ*g/mL ICG, cell viability was almost the same between the cultures in the dark and under illumination. In the cultures with ICG in the dark, cell viability and cell death were not significantly changed at any concentrations of ICG (Figures 2(a) and 2(b), black bars). On the other hand, cell viability decreased and the cell death rate increased in a dose-dependent manner in the cultures with more than 2.5 *μ*g/mL ICG under illumination (Figures 2(a) and 2(b), white bars).

Next, we observed morphological changes of the cultures with or without 10 *μ*g/mL ICG after a 24-hour incubation in the dark or under illumination (Figures 2(c)–2(f)). In the culture with ICG under illumination, almost all of the cells changed to be oval with a distinct cellular boundary, although most of the cells in other culture conditions maintained a flattened morphology. Subsequently, we performed PI staining and TUNEL to determine whether apoptosis was involved in cell death (Figures 2(g)–2(j)). While a few TUNEL-positive cells were observed in cultures with or without ICG in the dark, or in cultures without ICG under illumination (Figures 2(g)–2(j)), TUNEL- and/or PI-positive cells increased predominantly in cultures with ICG under illumination (Figure 2(j)). The number of PI- and TUNEL-positive cells cultured with ICG under illumination was significantly increased compared with that in other experimental conditions (Figures 2(k) and 2(l)).

### 2.3. Production of Intracellular Reactive Oxygen Species and Lipid Peroxidation by ICG Exposure and/or Illumination

**Figure 3:**
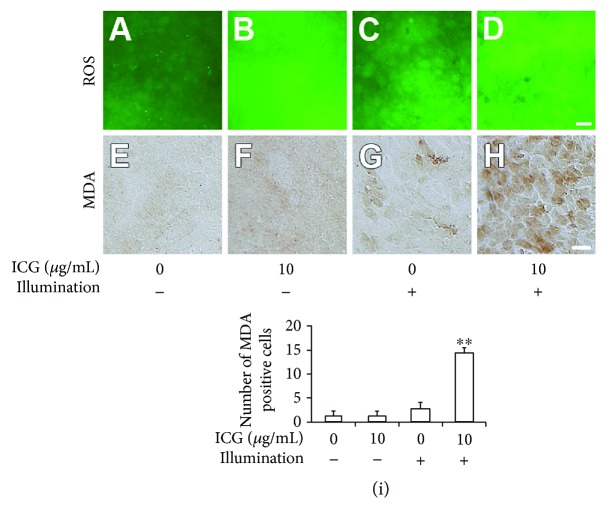
Production of reactive oxygen species and lipid peroxidation by ICG exposure and/or illumination. (a–d) Fluorescence images of reactive oxygen species formation in cultures with 0 or 10 *μ*g/mL ICG in the dark or without illumination. Scale bar: 50 *μ*m. (e–h) Bright-field micrographs of malondialdehyde (MDA) immunostaining in cells after a 24-hour incubation with or without 10 *μ*g/mL ICG cultured in the dark or under illumination. Scale bar: 25 *μ*m. (i) Quantitation of MDA-positive cells in 10,000 *μ*m^2^. At least four squares of each experiment were analyzed. Dunnett's test: ^∗∗^*P* < 0.01.

To investigate the participation of oxidative stress by ICG exposure and/or illumination, we examined the production of ROS in the cultures with or without 10 *μ*g/mL ICG in the dark or under illumination (Figures 3(a)–3(d)). Furthermore, we detected malondialdehyde (MDA) as a marker of lipid peroxidation in the cultures after a 24-hour incubation (Figures 3(e)–3(h)) [30, 31]. In the culture with ICG in the dark, ROS signals were increased uniformly and MDA staining was mildly increased uniformly compared with the control (Figures 3(b) and 3(f)). In the culture without ICG under illumination, the features of ROS signals and MDA staining were similar with those of the control but the intensities of ROS signals and MDA staining were enhanced (Figures 3(c) and 3(g)). In the culture with ICG under illumination, the intensities of ROS signals and MDA staining were increased entirely and enhanced irregularly in each cell (Figures 3(d) and 3(h)). The number of MDA-positive cells cultured with ICG under illumination was significantly increased compared with that in other experimental conditions of cultures (Figure 3(i)).

### 2.4. Changes of Absorption Spectra of ICG by Cell and/or Illumination and ICG Staining on Cells

**Figure 4:**
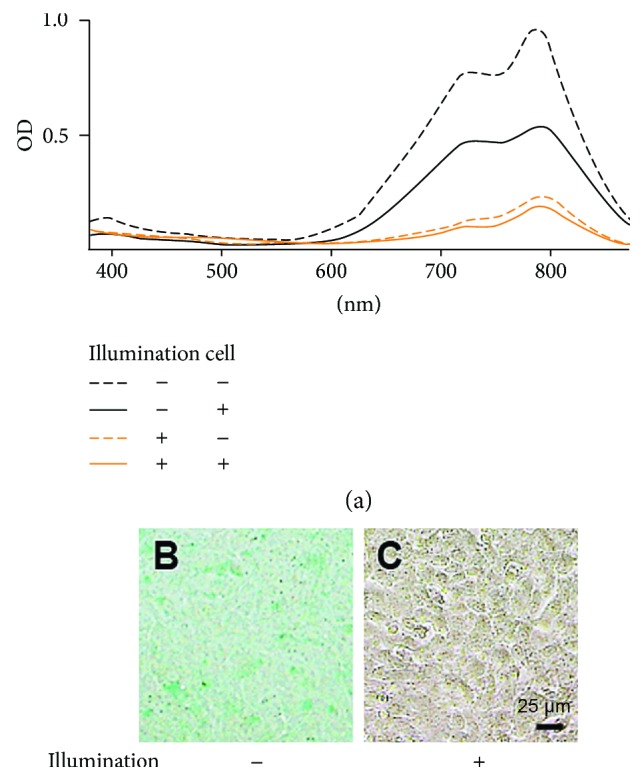
Changes of absorption spectra of ICG cultured with or without cells and/or illumination. (a) Absorption spectra of PBS-based media containing 10 *μ*g/mL ICG after a 24-hour incubation with or without cells cultured in the dark or under illumination. (b, c) Bright-field micrographs in cells cultured with or without 10 *μ*g/mL ICG in the dark or under illumination.

We also examined the absorption spectrum of 10 *μ*g/mL ICG under illumination after 24 hours of incubation (Figure 4(a)). The absorption spectrum of the medium incubated without cells in the dark as a control (Figure 4(a), black dashed line) showed a convex curve with two peaks at 728 nm (primary peak) and 786 nm (secondary peak). In the medium incubated with cells in the dark (black bold line), the primary and secondary peak absorbance reduced to approximately 61% and 56% compared to the control, respectively. In the medium incubated with or without cells under illumination (orange lines), the primary and secondary peak absorbance reduced to approximately 17% and 24% in the medium without cells (orange dashed line) and approximately 13% and 19% in the medium incubated with cells (orange bold line), respectively. These results indicated that the absorption spectrum of 10 *μ*g/mL ICG decreased by incubation with cells and is further reduced under illumination, but is similar under illumination with or without cells. In addition, the cells incubated with ICG in the dark were stained green (Figure 4(b)), while the cells incubated with ICG under illumination were not stained on bright-field microscopy (Figure 4(c)).

### 2.5. Cytotoxicity of Preilluminated Medium Containing ICG

**Figure 5:**
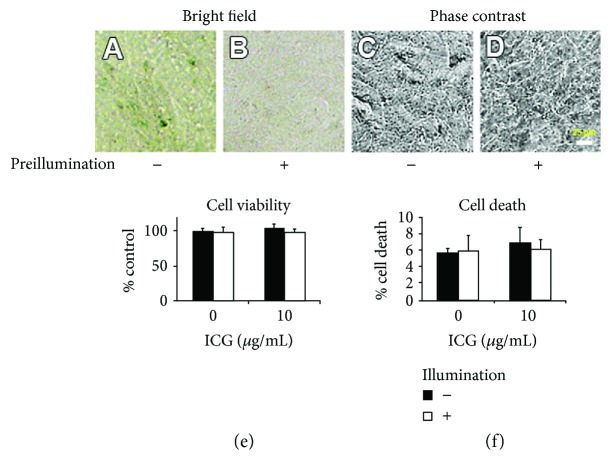
Staining ability and cytotoxicity of the preincubated medium with ICG in the dark or under illumination. (a, b) Bright-field micrographs and (c, d) phase-contrast micrographs of cultures in the preincubated medium containing 10 *μ*g/mL ICG in the dark or under illumination after 24 hours. (e, f) Cell viability and cell death rate in the preincubated medium containing 0 or 10 *μ*g/mL ICG after a 24-hour culture. Cell viability of each culture was normalized to that of culture in the preincubated medium with 0 *μ*g/mL ICG in the dark. There was no significant difference in cell viability and cell death rate among all conditions of cultures.

To examine whether ICG was changed to be cytotoxic under continuous irradiation of ambient light, the PBS-based medium containing 10 *μ*g/mL ICG was incubated in the dark or under illumination for 24 hours, and the cells were cultured in preincubated media for 24 hours in the dark. Intensity of ICG staining on the cells (Figures 5(a) and 5(b)) and cell morphology (Figures 5(c) and 5(d)) were observed by bright-field microscopy and phase-contrast microscopy, respectively. The cells cultured in the preincubated medium in the dark were stained green uniformly, and the cells cultured in the preincubated medium under illumination showed faint staining of green, since the absorption spectrum of 10 *μ*g/mL ICG decreased by incubation under illumination (Figure 4(a)). However, the cells cultured both in the preincubated media in the dark and under illumination maintained a flattened morphology. The preincubated medium with ICG both in the dark and under illumination had no significant effect in cell viability and cell death (Figures 5(e) and 5(f)). Cell viability and cell death rate of each culture were normalized to those of the culture in the preincubated medium without ICG.

### 2.6. Cytoprotective Effects of Blocking Green to Red Light against ICG Phototoxicity

**Figure 6:**
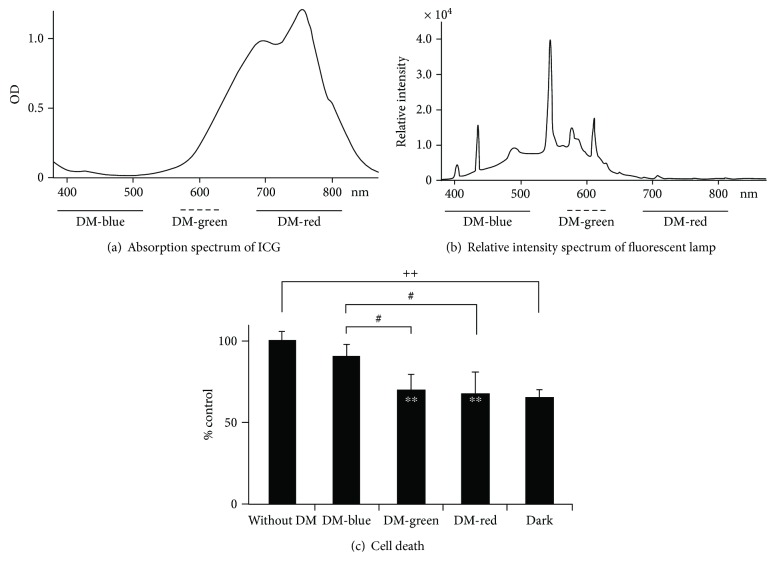
Mitigating effect of blocking green to red light against ICG phototoxicity. (a) Absorption spectrum of PBS-based medium with 10 *μ*g/mL ICG. Solid, dotted, or bold bar under the horizontal axis indicates wavelength ranges in which DM-blue, DM-green, and DM-red block wavelengths correspond to each DM color, respectively. (b) Relative intensity spectrum of light from the fluorescent lamp. (c) Cell death rates in cultures with 10 *μ*g/mL ICG in the dark and under illumination with or without DM after a 24-hour incubation. The cell death rate in each culture was normalized to that of culture under illumination without DM. DM: dichroic mirror. Dunnett's test: ^∗∗^*P* < 0.01, unpaired Student's *t*-test: ^++^*P* < 0.01, Student-Newman-Keuls test: ^#^*P* < 0.05.

Subsequently, we investigated the preventive effects of blocking specific light wavelengths in visible rays against ICG phototoxicity. The absorption spectrum of 10 *μ*g/mL ICG in the PBS-based medium had two peaks at 697 nm and 755 nm (Figure 6(a)). The relative intensity spectrum of light from the fluorescent lamp showed a multipeaked curve (Figure 6(b)). The interrupted wavelength range by DM-blue, DM-green, or DM-red was 395 nm to 516 nm (violet to blue), 556 nm to 616 nm (green to orange), and 641 nm to 860 nm (red to infrared), respectively (Figures 6(a) and 6(b)). The total photoenergy exposed to the cultures under illumination without DM and with DM-blue, DM-green, or DM-red for the 24-hour incubation was 76.3, 46.7, 44.9, and 56.2 J/cm^2^, respectively. The cell death rate of the cultures under illumination was reduced by DM-green and DM-red compatible with that in the cultures in the dark, but not by DM-blue (Figure 6(c)). The amount of cell death in each culture was normalized to that of the culture under illumination without DM.

## 3. Discussion

The present study was designed to investigate the potential phototoxicity of ICG which continuously contacts RPE cells under continuous illumination mimicking chorioretinal conditions after ICG angiography in the clinical setting and to examine what sorts of blocking of light wavelengths were cytoprotective against ICG phototoxicity. In this study, we demonstrated that ICG at prospective residual concentrations in the retina and choroid after ICG angiography would have potential phototoxicity via oxidative damage under irradiation of ambient light in an in vitro model and that blocking green to red light overlapping absorption spectra of ICG could prevent its phototoxicity.

First, we selected an appropriate medium for the present study. Several culture media have been used to investigate cytotoxicity of photosensitizers under irradiation of visible rays *in vitro* [19, 29, 31, 32]. In an illuminated culture, a culture medium must be transparent for visible rays, resistant to denaturation by light irradiation, and capable of maintaining cell viability for the indicated experiment periods. The PBS-based medium satisfied the abovementioned requirements in our experimental conditions.

The color temperature of fluorescent lamp light and the intensity of illumination on the cells were considerable factors in this study. The International Commission on Illumination (the CIE) has introduced well-known standard illuminants in the categories of illuminant A to F [33]. The CIE Standard illuminant D65 in illuminant D represents natural daylight (6504 K) and is one of the standard illuminations used in color science and engineering. The illuminant series F represents various types of fluorescent lighting (6500 K) emitting full-spectrum light similarly with illuminant D65. Regarding intensity of illumination, the intensities of fluorescent lamp light between 500 lx and 15,000 lx were adopted as intensities of ambient light *in vivo* [34–36]. We also adopted 2000 lx as an intensity of ambient light *in vitro* [19, 29]. Based on previous reports, we applied 2000 lx on the cells under a fluorescent lamp belonging to the illuminant series F as an experimental condition imitating irradiation of ambient light.

In the ICG clearance test, the loading dose is generally 0.5 mg/kg and the concentration of ICG in the blood just after ICG angiography is approximately 10 *μ*g/mL in healthy subjects [7, 9]. ICG is eliminated quickly from the circulating blood, and the residual concentrations of ICG in the blood after 15 minutes of the angiography decreased to be under 10% in healthy subjects with normal liver function [7, 9]. On the other hand, the residual concentration of ICG in retinal pigment epithelium-Bruch's membrane complex after 24 hours of the angiography was mostly 0.05 *μ*g/mL in young normal eyes, aged normal eyes, and eyes with age-related macular degeneration [9]. However, the practical residual concentrations of ICG in the retina and choroid after ICG angiography remain to be unclear because the concentrations of ICG in the retina and choroid would depend in large part on general conditions such as renal insufficiency and hepatic insufficiency as well as chorioretinal diseases which break the blood-retina barrier and allow leakage and pooling of ICG in the retina and choroid [13–15]. Thus, we chose 24-hour continuous exposure of ICG at the concentrations from 0.5 to 10 *μ*g/mL as prospective residual concentrations of ICG after ICG angiography.

In incubations with ICG under illumination, cell viability was decreased and cell death was increased significantly in continuous exposure of more than 5.0 *μ*g/mL ICG. The level of intracellular ROS was elevated, and formation of MDA was remarkably increased in the culture with ICG under illumination. Oxidative damages by singlet oxygen via type II photooxidation and toxicity of light-induced ICG decomposition products have been known as factors of ICG phototoxicity [26, 28]. In the present experimental conditions according to the clinical setting of ICG angiography, the preilluminated ICG-containing medium did not demonstrate remarkable cytotoxicity which is different from the previous report regarding the cytotoxicity of decomposition products of preilluminated ICG [28]. Therefore, it is suggested that oxidative damages via type II photooxidation may be a critical mechanism of phototoxicity induced in ICG angiography.

ICG is incorporated to RPE cells via a Na^+^-involved cotransporter and is retained in the cytoplasm [25, 37]. In the present study, the ICG absorption spectrum was reduced in the cultures with cells under illumination, but was equal to the cultures without cells under illumination (Figure 4(a)). In addition, the cells were not stained green in the cultures with ICG under illumination (Figures 4(b) and 4(c)). LDH leakage is a marker of cell membrane damage by stimulation of illumination, and the RPE cells incubated with ICG in the dark were stained green (Figure 4(b)) [38]. Therefore, it is possible that since ICG molecules could pass freely through damaged cell membranes, intracellular ICG in the RPE cells under illumination would be extracted.

The wavelength of ambient light overlaps with that of ICG absorbance, and ambient light induces phototoxicity of ICG [2, 24, 28]. The present study demonstrates that blocking of green or red light wavelength prevents ICG phototoxicity in RPE cells under continuous illumination, but blocking of blue light does not. The total photoenergy exposed to the cultures under illumination with DM-blue was 46.7 J/cm^2^, which was higher than 44.9 J/cm^2^ with DM-green but was lower than 56.2 J/cm^2^ with DM-red for the 24-hour incubation. Since the photoenergy for wavelengths from 380 to 430 nm involved in the wavelengths partially blocked by DM-blue could not be detected by the radiometer used in this study, the photoenergy within this range was not counted. Therefore, the total photoenergy of the culture blocked with DM-blue should be relatively overestimated compared with that of DM-green or DM-red. Although total photoenergy of the culture with DM-red was higher than that of the culture with DM-blue, the cell death rate of blocking red light could be suppressed compared with that of blocking blue light, suggesting that cytoprotective effects using DMs were not induced by reducing total photoenergy on the RPE cells, but were by reducing the photoenergy in specific wavelengths overlapping predominant absorbance of ICG.

The present study has several major limitations. First, ARPE-19 cells are cultured human RPE cells and are not well differentiated enough to express pigmentation and all RPE markers. Further *in vitro* experiments using cultured human foetal RPE cells and/or human iPS-derived RPE cells which could have differentiation ability of expressing pigmentation and various tight junctions are required to allow a better understanding of the potential phototoxicity of ICG [39, 40]. Second, the results of our study to reveal the potential phototoxicity of ICG were limited in *in vitro* settings. In the future, we would perform further examinations to confirm the phototoxic effects of ICG *in vivo* models using pigmented animals.

In conclusion, this study investigated the phototoxicity of ICG on human RPE cells at the levels of residual ICG after angiography under ambient light. We found that ICG at the estimated intravenous concentrations after ICG angiography induced the potential phototoxicity on human RPE cells via oxidative damage under ambient light and that the cytotoxicity was reduced by blocking green to red light wavelengths. This suggests that wearing shading devices such as sunglasses may be recommended to prevent ICG phototoxicity in the retina and choroid after the angiography for a short period, especially under the sun.

## 4. Methods

### 4.1. Cell Culture and Medium for Illuminated Culture

The human RPE cell line ARPE-19 was obtained from the American Type Culture Collection (Manassas, VA). The cells were cultured in DMEM/F12 (Sigma-Aldrich, Poole, UK) containing 10% foetal bovine serum (FBS; JRH Biosciences, Lenexa, KS) and antibiotics (100 U/mL penicillin G and 100 *μ*g/mL streptomycin sulfate; Sigma-Aldrich) at 37°C in a humidified atmosphere of 5% CO_2_ and 95% air. The cells were trypsinized and subcultured to confluence in multiwell polystyrene plates.

To eliminate shielding effects of coloration in the culture medium, we prepared a colorless PBS-based medium consisting of Dulbecco's phosphate-buffered saline (PBS; Sigma-Aldrich) supplemented with 1% FBS, 1 mg/mL glucose, 1 mg/mL CaCl_2_, 1 mg/mL MgCl_2_, and antibiotics for a culture under continuous illumination by a fluorescent lamp [19, 29].

### 4.2. Culture with ICG under Continuous Fluorescent Lamp Illumination

ICG (Daiichi Sankyo, Tokyo, Japan) was dissolved in distilled water to a final concentration of 25 mg/mL. The ICG solution was further diluted with the PBS-based medium to achieve final ICG concentrations of 0.5, 1.0, 2.5, 5.0, and 10 *μ*g/mL for experiments. The cells in the confluent monolayer were washed once with PBS and then cultured in the PBS-based medium with or without ICG either in the dark (within a metal box) or under 2000 lux illumination from a daylight-colored fluorescent lamp (6500 K, Sunline; Hitachi, Tokyo, Japan) for 24 hours at 37°C in a humidified air in an incubator fitted with the fluorescent lamp (CPO_2_-171, Hirasawa, Tokyo, Japan).

For blocking of specific light wavelengths, the cells were cultured in 4-well plates. A blue, green, or red dichroic mirror (DM; 4 × 4 cm^2^, Koshin, Tokyo, Japan) was attached on top of the culture plate. Each dichroic mirror reflects wavelengths corresponding to each color and allows wavelengths of other colors to pass through. The sides of the plates were covered with aluminum foil to prevent transmission of light from the fluorescent lamp. The cells were cultured for 24 hours in the PBS-based medium containing 10 *μ*g/mL ICG in the dark or under illumination with or without a DM.

### 4.3. Culture in Preincubated Medium Containing ICG

The PBS-based medium containing 10 *μ*g/mL ICG was incubated in the dark or under illumination for 24 hours, and then the cells were cultured in the preincubated media in the dark for 24 hours.

### 4.4. Measurements of Cell Viability and Cell Death Rate

Quantitative assessment of cell viability was evaluated by measuring mitochondrial reductase activity using the 3-(4,5-dimethylthiazol-2-yl)-5-(3-carboxymethoxyphenyl)-2-(4-sulfophenyl)-2H-tetrazolium, inner salt (MTS) assay (Promega, Madison, WI). Quantitative assessment of cell death was performed using a lactate dehydrogenase assay (LDH; Roche, Mannheim, Germany) which measures the activity of LDH released from dead cells into culture supernatant. After collecting an aliquot of culture supernatant to measure the amount of LDH from dead cells, the cells were lysed with 1% Triton X-100 to release intracellular LDH from surviving cells. Cell death rate was calculated as follows: supernatant LDH activity divided by total (supernatant + lysed cells) LDH activity × 100% [29]. MTS assay and LDH assay were performed according to the manufacturers' protocols.

### 4.5. Detection of Apoptosis

Apoptosis was detected by terminal deoxynucleotidyl transferase-mediated dUTP nick-end labeling (TUNEL) using fluorescein-conjugated deoxy UTP as the substrate (In Situ Cell Death Detection Kit; Roche) according to the manufacturer's protocols. In some experiments, propidium iodide (PI) was added to detect dead cells. The samples were observed and photographed with a universal microscope (Biozero; Keyence, Osaka, Japan).

### 4.6. Detection of Intracellular Reactive Oxygen Species

Total intracellular reactive oxygen species (ROS) in live cells were detected using the ROS/RNS Detection Kit (Enzo Life Sciences Inc., Farmingdale, NY) according to the manufacturer's instructions. The cells were loaded with the reagent for 2 hours at 37°C in DMEM/F12 containing 10% FBS. After the treatment, the culture medium was changed to the colorless medium. Subsequently, the cells with 0 or 10 *μ*g/mL ICG were cultured in the dark or under illumination for 30 minutes. The fluorescence of 2′,7′-dichlorodihydrofluorescein exhibiting intracellular ROS level in each culture was detected by fluorescence microscopy through a green filter (maximal excitation/maximal emission wavelengths: 490/525 nm).

### 4.7. Detection of Lipid Peroxidation

The cells were fixed with 10% neutralized formalin. Malondialdehyde (MDA) as a degraded product of polyunsaturated lipids by ROS was detected by immunostaining of dihydropyridine lysine adducts [30, 31]. The fixed cells were reacted with anti-MDA mouse antibody (JaICA, Shizuoka, Japan) and stained by the avidin-biotin-peroxidase complex method (Vector Laboratories, Burlingame, CA) with diaminobenzidine according to the manufacturer's protocols.

### 4.8. Spectrophotometric Measurement

The PBS-based medium containing 10 *μ*g/mL ICG and the cells with 10 *μ*g/mL ICG in the dark or under illumination were incubated for 24 hours. Absorption spectra of the medium and culture supernatants of the cells were measured using a spectrophotometer (U-0080D; Hitachi, Tokyo, Japan).

### 4.9. Spectrum of Fluorescent Lamp Light and Total Photoenergy on Cells

The relative intensity spectrum of the fluorescent lamp light was measured using a spectroscope (USB4000; Ocean Photonics, Tokyo, Japan). The total photoenergy on the cells in wavelengths from 430 nm to 1000 nm was evaluated using a radiometer (PD300-BB; Ophir Japan, Saitama, Japan) [29].

### 4.10. Statistical Analysis

Four samples in each group were measured for each experiment. Data in graphs are expressed as means with standard deviations. Data of over three groups were analyzed by nonrepeated measures ANOVA with Dunnett's test for comparison with control. Unpaired two-tailed Student's *t*-test was used to analyze data of two groups, and the Student-Newman-Keuls test was used in multiple comparisons. *P* < 0.05 was considered to be significant.

## Data Availability

The data used to support the findings of this study are available from the corresponding author upon request.

## References

[1] Landsman M. L., Kwant G., Mook G. A., Zijlstra W. G. (1976). Light-absorbing properties, stability, and spectral stabilization of indocyanine green. *Journal of Applied Physiology*.

[2] Haritoglou C., Gandorfer A., Schaumberger M., Tadayoni R., Gandorfer A., Kampik A. (2003). Light-absorbing properties and osmolarity of indocyanine-green depending on concentration and solvent medium. *Investigative Ophthalmology & Visual Science*.

[3] Benson R. C., Kues H. A. (1978). Fluorescence properties of indocyanine green as related to angiography. *Physics in Medicine and Biology*.

[4] Kogure K., David N. J., Yamanouchi U., Choromokos E. (1970). Infrared absorption angiography of the fundus circulation. *Archives of Ophthalmology*.

[5] Flower R. W., Hochheimer B. F. (1973). A clinical technique and apparatus for simultaneous angiography of the separate retinal and choroidal circulations. *Investigative Ophthalmology & Visual Science*.

[6] Cherrick G. R., Stein S. W., Leevy C. M., Davidson C. S. (1960). Indocyanine green: observations on its physical properties, plasma decay, and hepatic extraction. *The Journal of Clinical Investigation*.

[7] Namihisa T., Nambu K. (1980). The chemical examination of blood and urine. ICG and BSP. How shall we judge the result (in Japanese)?. *Japanese Journal of Clinical Medicine*.

[8] Benya R., Quintana J., Brundage B. (1989). Adverse reactions to indocyanine green: a case report and a review of the literature. *Catheterization and Cardiovascular Interventions*.

[9] Mori K., Gehlbach P. L., Nishiyama Y., Deguchi T., Yoneya S. (2002). The ultra-late phase of indocyanine green angiography for healthy subjects and patients with age-related macular degeneration. *Retina*.

[10] Yoneya S. (2004). A new approach for studying the retinal and choroidal Circulation. *Nippon Ganka Gakkai Zasshi*.

[11] Chang A. A., Morse L. S., Handa J. T. (1998). Histologic localization of indocyanine green dye in aging primate and human ocular tissues with clinical angiographic correlation. *Ophthalmology*.

[12] Chang A. A., Zhu M., Billson F. A., Kumar N. L., Beaumont P. E. (2004). Indocyanine green localisation in surgically excised choroidal neovascular membrane in age related macular degeneration. *The British Journal of Ophthalmology*.

[13] Lambert N. G., ElShelmani H., Singh M. K. (2016). Risk factors and biomarkers of age-related macular degeneration. *Progress in Retinal and Eye Research*.

[14] Yonekawa Y., Miller J., Kim I. (2015). Age-related macular degeneration: advances in management and diagnosis. *Journal of Clinical Medicine*.

[15] Baltmr A., Lightman S., Tomkins-Netzer O. (2016). Vogt–Koyanagi–Harada syndrome – current perspectives. *Clinical Ophthalmology*.

[16] Rodrigues E. B., Maia M., Meyer C. H., Penha F. M., Dib E., Farah M. E. (2007). Vital dyes for chromovitrectomy. *Current Opinion in Ophthalmology*.

[17] Engelbrecht N. E., Freeman J., Sternberg P. (2002). Retinal pigment epithelial changes after macular hole surgery with indocyanine green-assisted internal limiting membrane peeling. *American Journal of Ophthalmology*.

[18] Haritoglou C., Gandorfer A., Gass C. A., Schaumberger M., Ulbig M. W., Kampik A. (2002). Indocyanine green-assisted peeling of the internal limiting membrane in macular hole surgery affects visual outcome: a clinicopathologic correlation. *American Journal of Ophthalmology*.

[19] Sato T., Ito M., Ishida M., Karasawa Y. (2010). Phototoxicity of Indocyanine green under continuous fluorescent lamp illumination and its prevention by blocking red light on cultured Müller cells. *Investigative Ophthalmology & Visual Science*.

[20] Liu Z., Meyer C. H., Fimmers R., Stanzel B. V., International Chromovitrectomy Collaboration (2014). Indocyanine green concentrations used in chromovitrectomy cause a reversible functional alteration in the outer blood-retinal barrier. *Acta Ophthalmologica*.

[21] Ejstrup R., La Cour M., Heegaard S., Kiilgaard J. F. (2012). Toxicity profiles of subretinal indocyanine green, Brilliant Blue G, and triamcinolone acetonide: a comparative study. *Graefe's Archive for Clinical and Experimental Ophthalmology*.

[22] Gandorfer A., Haritoglou C., Gandorfer A., Kampik A. (2003). Retinal damage from indocyanine green in experimental macular surgery. *Investigative Ophthalmology & Visual Science*.

[23] Ho J. D., Tsai R. J., Chen S. N., Chen H. C. (2003). Cytotoxicity of indocyanine green on retinal pigment epithelium: implications for macular hole surgery. *Archives of Ophthalmology*.

[24] Rodrigues E. B., Meyer C. H., Mennel S., Farah M. E. (2007). Mechanisms of intravitreal toxicity of indocyanine green dye: implications for chromovitrectomy. *Retina*.

[25] Chang A. A., Zhu M., Billson F. (2005). The interaction of indocyanine green with human retinal pigment epithelium. *Investigative Ophthalmology & Visual Science*.

[26] Baumler W., Abels C., Karrer S. (1999). Photo-oxidative killing of human colonic cancer cells using indocyanine green and infrared light. *British Journal of Cancer*.

[27] Kernt M., Hirneiss C., Wolf A. (2012). Indocyanine green increases light-induced oxidative stress, senescence, and matrix metalloproteinases 1 and 3 in human RPE cells. *Acta Ophthalmologica*.

[28] Engel E., Schraml R.¨d., Maisch T. (2008). Light-induced decomposition of indocyanine green. *Investigative Ophthalmology & Visual Science*.

[29] Takayama K., Sato T., Karasawa Y., Sato S., Ito M., Takeuchi M. (2012). Phototoxicity of indocyanine green and Brilliant Blue G under continuous fluorescent illumination on cultured human retinal pigment epithelial cells. *Investigative Ophthalmology & Visual Science*.

[30] Del Rio D., Stewart A. J., Pellegrini N. (2005). A review of recent studies on malondialdehyde as toxic molecule and biological marker of oxidative stress. *Nutrition, Metabolism, and Cardiovascular Diseases*.

[31] Chu R., Zheng X., Chen D., Hu D. N. (2006). Blue light irradiation inhibits the production of HGF by human retinal pigment epithelium cells in vitro. *Photochemistry and Photobiology*.

[32] Lornejad-Schafer M. R., Lambert C., Breithaupt D. E., Biesalski H. K., Frank J. (2007). Solubility, uptake and biocompatibility of lutein and zeaxanthin delivered to cultured human retinal pigment epithelial cells in tween40 micelles. *European Journal of Nutrition*.

[33] Schanda J. (2007). *Colorimetry*.

[34] Ashby R., Ohlendorf A., Schaeffel F. (2009). The effect of ambient illuminance on the development of deprivation myopia in chicks. *Investigative Ophthalmology & Visual Science*.

[35] Ashby R. S., Schaeffel F. (2010). The effect of bright light on lens compensation in chicks. *Investigative Ophthalmology & Visual Science*.

[36] Kamoshita M., Toda E., Osada H. (2016). Lutein acts via multiple antioxidant pathways in the photo-stressed retina. *Scientific Reports*.

[37] Ho J. D., Chen H. C., Chen S. N., Tsai R. J. (2004). Reduction of indocyanine green-associated photosensitizing toxicity in retinal pigment epithelium by sodium elimination. *Archives of Ophthalmology*.

[38] Wielgus A. R., Zhao B., Chignell C. F., Hu D. N., Roberts J. E. (2010). Phototoxicity and cytotoxicity of fullerol in human retinal pigment epithelial cells. *Toxicology and Applied Pharmacology*.

[39] Hu J., Bok D. (2001). A cell culture medium that supports the differentiation of human retinal pigment epithelium into functionally polarized monolayers. *Molecular Vision*.

[40] Greene W. A., Muñiz A., Plamper M. L., Kaini R. R., Wang H.-C. (2014). MicroRNA expression profiles of human iPS cells, retinal pigment epithelium derived from iPS, and fetal retinal pigment epithelium. *Journal of Visualized Experiments*.

